# Sporoderm-removed ganoderma lucidum spore powder (S-GLSP) alleviates neuroinflammation injury by regulating microglial polarization through inhibition of NLRP3 inflammasome activation

**DOI:** 10.3389/fphar.2025.1690192

**Published:** 2025-11-25

**Authors:** Wenli Li, Wei Huang, Peng Zhou, Yongchuan Yao, Biao Cai, Shu Ye

**Affiliations:** 1 School of Integrated Chinese and Western Medicine, Anhui University of Chinese Medicine, Hefei, China; 2 Anhui Provincial Key Laboratory of Chinese Medicinal Formula, Hefei, China; 3 Innovation Center for Medical Research on the Prevention and Treatment of Neurodegenerative Diseases Using Integrated Traditional Chinese and Western Medicine, Hefei, China; 4 The First Affiliated Hospital of Anhui University of Traditional Chinese Medicine, Hefei, China

**Keywords:** sporoderm-removed ganoderma lucidum spore powder, Alzheimer’s disease, microglial polarization, neuroinflammation, NLRP3 inflammasome

## Abstract

**Introduction:**

Sporoderm-Removed Ganoderma lucidum Spore Powder (S-GLSP), derived from the spores of the medically valued fungus Ganoderma lucidum, exhibits diverse pharmacological activities and shows considerable potential in the treatment of Alzheimer’s disease (AD). However, its underlying mechanisms of action remain incompletely elucidated. This study aims to investigate the protective effects of S-GLSP against AD and to explore the molecular mechanisms involved.

**Materials and Methods:**

The chemical profile of S-GLSP extract was characterized using LC-MS/MS. Alzheimer’s disease models were established both *in vivo* and *in vitro*: a rat model was induced by D-galactose combined with intracerebroventricular injection of Aβ, while a cellular model was stimulated with LPS. The neuroprotective effects of S-GLSP were assessed through behavioral tests and hematoxylin-eosin (HE) staining. Immunofluorescence staining, Western blot (WB), RT-qPCR, and ELISA were employed to evaluate microglial polarization and NLRP3 inflammasome activation. Cell viability was measured using MTT and EdU assays. Finally, NLRP3 knockdown was performed to verify whether S-GLSP modulates microglial polarization via regulation of the NLRP3 inflammasome.

**Results:**

A total of 42 chemical compounds were identified in S-GLSP, including flavonoids, alkaloids, terpenoids, saccharides, phenolics, fatty acids, nucleosides, amino acids, and other. S-GLSP treatment alleviated neuronal damage, improved learning and memory deficits, and reduced the expression of phosphorylated tau (p-tau) in AD model rats. Further experiments *in vitro* and *in vivo* showed that S-GLSP downregulated M1 phenotypic markers (CD86, iNOS, TNF-α) and upregulated M2 markers (CD206, Arg-1, IL-10). Moreover, S-GLSP inhibited NLRP3 inflammasome activation and regulated the secretion of IL-1β and IL-18, effects that were consistent with those observed following NLRP3 knockdown.

**Conclusion:**

Our findings demonstrate that S-GLSP alleviates Alzheimer’s disease pathology by inhibiting NLRP3 inflammasome activation, promoting a shift in microglial polarization from the M1 to the M2 phenotype, and modulating the release of inflammatory cytokines. This study provides novel mechanistic insights into the therapeutic potential of S-GLSP for AD.

## 1 Introduction

Alzheimer’s disease (AD) remains one of the most challenging progressive neurodegenerative disorders, characterized by memory loss, cognitive decline, and impairments in reasoning and emotional regulation. As the disease advances, patients gradually lose the ability to live independently, imposing substantial societal and familial burdens (Knopman et al., 2021). To date, drug development for AD has progressed slowly, and existing approved medications are unable to reverse the pathological course of the disease. Notably, recent therapeutic strategies have shifted toward targeting neuroimmune cells—such as microglia and astrocytes—and modulating peripheral inflammation to alleviate central nervous system (CNS) inflammation, offering promising diagnostic and therapeutic avenues for AD (Yan et al., 2024). Among these, microglia-mediated chronic neuroinflammation has been shown to drive AD progression by promoting amyloid-beta (Aβ) plaque accumulation and neurofibrillary tangle (NFT) formation (Kwon and Koh, 2020). Therefore, reprogramming aberrant microglial polarization has emerged as a potential strategy to curb disease progression. In this context, multi-target compounds derived from Traditional Chinese Medicine have attracted increasing research attention.

Ganoderma lucidum (Curtis) P. Karst, a medicinal and edible fungus widely used in Asian countries, represents a botanical drug with substantial therapeutic value. Systematic studies have revealed that Ganoderma lucidum and its active compounds exhibit considerable potential in AD treatment. They are known to delay disease progression and improve cognitive function through multiple mechanisms, including inhibition of tau hyperphosphorylation, reduction of Aβ aggregation, suppression of neuronal apoptosis, regulation of acetylcholinesterase, modulation of microglial activation, and interference with the NF-κB/MAPK signaling pathway (Chen et al., 2024). Ganoderma lucidum spore powder (GLSP), containing the full spectrum of bioactive constituents of the fungus, comprises a wide range of chemical compounds such as triterpenoids, polysaccharides, fatty acids, alkaloids, and vitamins, which collectively contribute to its anti-inflammatory, immunomodulatory, antioxidant, and neuroprotective properties (Zhang et al., 2024). Sporoderm-removed Ganoderma lucidum spore powder (S-GLSP), a highly processed formulation, exhibits improved bioavailability and higher concentrations of active compounds (Soccol et al., 2016). In 2020, broken Ganoderma spores were officially listed in China’s Health Food Catalog (The State Pharmacopoeia Commission of People’s Republic of China, 2020). A previous study reported that S-GLSP extract significantly improved memory performance in a rat model of Alzheimer’s induced by intracerebroventricular streptozotocin (Zhao et al., 2021).

Nevertheless, the protective role of S-GLSP in AD and its underlying molecular mechanisms remain incompletely understood. The NOD-like receptor protein 3 (NLRP3) inflammasome—composed of NLRP3, ASC, and pro-caspase-1 (Fu and Wu, 2023)—is the most widely studied inflammasome complex and is closely linked to AD pathogenesis (Li and Gong, 2025). Upon activation by Aβ, microglia promote the assembly of the NLRP3 inflammasome, which in turn triggers a cascade of inflammatory responses (Heneka et al., 2013; Van Zeller et al., 2021). Excessive NLRP3 activation not only exacerbates tau pathology and synaptic dysfunction but also sustains a harmful inflammatory milieu via elevated IL-1β release (Felsky et al., 2019). Thus, microglial NLRP3 is considered a key node linking Aβ and tau pathology in AD (Ising et al., 2019).

In this study, we performed a comprehensive chemical profiling of S-GLSP using high-resolution mass spectrometry and evaluated its pharmacological effects in a rat model of AD induced by D-galactose and bilateral hippocampal Aβ_25-35_ injection. We further elucidated the mechanisms by which S-GLSP inhibits NLRP3-mediated neuroinflammation and regulates microglial polarization.

## 2 Materials and methods

### 2.1 Materials

S-GLSP (wall breaking rate ≥98%) were purchased from Anhui HuangshanYunle Ganoderma Lucidum Co., Ltd. (Anhui, China). Professor Nianjun Yu authenticated all G. lucidum at Anhui University of Chinese Medicine. D-galactose was obtained from Macklin (Shanghai, China). Aβ25-35 was acquired from Sigma-Aldrich (St. Louis, MO, United States).

### 2.2 Analysis of the chemical composition of S-GLSP

#### 2.2.1 Sample preparation and extraction

The S-GLSP (50 mg ± 2 mg) was weighed, mixed with beads, and 500 μL of extraction solution (MeOH: ACN: H_2_O, 2:2:1 v/v/v) containing deuterated internal standards. The mixture was incubated at −40 °C for 30 min. Then, the sample was centrifuged at 12,000 rpm (RCF = 13,800 × g, R = 8.6 cm) for 15 min at 4 °C. The supernatant was transferred to a fresh glass vial for analysis.

#### 2.2.2 LC-MS/MS analysis

LC-MS/MS analyses were performed using a UHPLC system (Vanquish, Thermo Fisher Scientific) equipped with a Phenomenex Kinetex C18 column (2.1 mm × 100 mm, 2.6 μm) coupled to an Orbitrap Exploris 120 mass spectrometer (Orbitrap MS, Thermo Fisher Scientific). Mobile phase A consisted of 0.01% acetic acid in water, and mobile phase B:IPA:ACN (1:1,v/v). The autosampler temperature was maintained at 4 °C, and the injection volume was 2 μL.

The Orbitrap Exploris 120 mass spectrometer was utilized for its capability to acquire MS/MS spectra in data-dependent acquisition (DDA) mode, controlled by the acquisition software Xcalibur (Thermo). The ESI source conditions were set as follows: sheath gas flow rate at 50 Arb, auxiliary gas flow rate at 15 Arb, capillary temperature at 320 °C, full MS resolution at 60,000, MS/MS resolution at 15,000, collision energy (SNCE) at 20/30/40, and spray voltage at 3.8 kV (positive mode) or −3.4 kV (negative mode), respectively.

#### 2.2.3 Data processing

The raw LC-MS/MS data were converted to the mzML format using MSConvert (ProteoWizard, version 3.0.18205). Data preprocessing was then performed using the XCMS package (version 4.7.3) in R (version 4.3.1), and Biotree DB (version 3.0).

### 2.3 Animals and treatments

Sprague-Dawley rats (250–350 g) were supplied by the Experimental Animal Center of Liaoning (Certificate No. SCXK (Liao) 2023-0001). The rats were housed individually in a temperature-controlled facility (23 °C ± 1 °C, 50%–60% humidity) under a 12 h/12 h light/dark cycle (lights on at 9:00 a.m., designated as ZT 0). Food and water were provided *ad libitum*. All animal experiments were conducted in accordance with the ARRIVE 2.0 guidelines (Boutron et al., 2020)and approved by the Animal Ethics Committee of Anhui University of Chinese Medicine (Approval No. AHUCM-rats-2023082).

**FIGURE 1:**
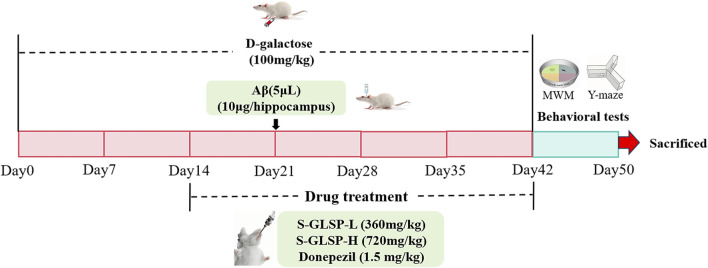
Timeline of the animal experimental protocol.

Fifty rats were randomly assigned to five groups: (n = 10 per group): control (CTRL), Model, low-dose S-GLSP (S-GLSP-L, 360 mg/kg), high-dose S-GLSP (S-GLSP-H, 720 mg/kg), and donepezil (1.5 mg/kg). The AD model was established as previously described (Huang et al., 2024). Briefly, rats received daily intraperitoneal injections of D-galactose (100 mg/kg) along with bilateral hippocampal injections of Aβ_25-35_ (10 μg per side). After 14 days of D-galactose administration, the S-GLSP-L and S-GLSP-H groups were treated orally with 360 mg/kg and 720 mg/kg S-GLSP (Zhao et al., 2021), respectively, while the DP group received 1.5 mg/kg of donepezil by gavage. Following the experimental procedures, rats were deeply anesthetized with sodium pentobarbital (45 mg/kg, i. p.) for terminal tissue collection. A schematic of the experimental timeline is provided in Figure 1.

### 2.4 Behavioral tests

All behavioral assessments were conducted by investigators blinded to the experimental groups. The tests were performed in a sequential manner: the Morris Water Maze (MWM) was conducted first, followed by the Y-Maze test after a 48-h rest period to minimize carryover effects between paradigms.

#### 2.4.1 Morris water maze

The spatial learning and memory abilities of rats were assessed using the Morris Water Maze Test. The maze consisted of a circular pool (160 cm in diameter) divided into four equal quadrants. A hidden escape platform was placed in the third quadrant, submerged 1 cm below the water surface. Days 1–4 (Acquisition Trial): rats were released sequentially from each of the four quadrants once daily and given 90 s to locate the hidden platform. Animals that failed to find the platform within the allotted time were gently guided to it and allowed to remain there for 15 s. Day 5 (Spatial Probe Test): rats were introduced into the pool from the first quadrant, and the time to reach the platform location in third quadrant within 90 s (escape latency) was recorded. Day 6 (Place Navigation Test): The platform was removed, and the rats were allowed to swim freely in the pool for 90 s. The number of crossings over the previous platform location was recorded. All behavioral sessions were video-tracked and analyzed using the SMART 3.0 system (Panlab, Barcelona, Spain). (The experiment was conducted under double-blind conditions.)

#### 2.4.2 Y maze

The Y-maze test was conducted using an apparatus with three identical arms (40 × 10 × 35 cm each), positioned at 120° angles and designated as the start arm, familiar arm, and novel arm. The experiment consisted of two phases separated by a 24-h interval. During the training phase, the novel arm was blocked, and rats were allowed to freely explore the start and familiar arms for 10 min. In the test phase conducted 24 h later, all three arms were made accessible, and each rat was allowed to explore the maze freely for 5 min. The time spent in the novel arm was recorded as an indicator of cortex-dependent working memory. All behavioral sessions were video-recorded and analyzed with the YMT-100 Y video tracking system (Chengdu Tai meng Software Co., Ltd., China). (The experiment was conducted under double-blind conditions.)

### 2.5 Hematoxylin & eosin (H&E) staining

After fixation with 4% paraformaldehyde, the brain tissue was dehydrated, cleared, and embedded in paraffin. The embedded paraffin blocks were sectioned into 5 μm-thick slices using microtome. Subsequently, the sections were stained with hematoxylin and eosin (H&E) for histological examination, and neuronal morphology in the hippocampal CA1 region was observed under a light microscope.

### 2.6 Western blot assays

The brain tissues or treated cells were lysed in RIPA lysis buffer (Beyotime Biotechnology, Shanghai, China) and protein concentrations were quantified by BCA assay. The protein samples were separated by 10%–12% SDS-PAGE, transferred to PVDF membranes. And the membranes were blocked with 5% skim milk at room temperature for 2 h, followed by an overnight incubation with primary antibodies: anti-phospho-tau (p-tau, 1:1,000, catalog no. 30505, Cell Signaling Technology, MA, United States), anti-iNOS (1:1,000, catalog no. 340668, ZenBio, Chengdu, China), anti-Arg-1 (1:1,000, catalog no. YM4860, Immunoway, Jiangsu, China), anti-NLRP3 (1:1,000, catalog no. ab263899, Abcam, Cambridge, United Kingdom), anti-ASC (1:1,000, catalog no. YT0365, Immunoway, Jiangsu, China), anti-cleaved Caspase-1 (1:1,000, catalog no. AF4005, Affinity, Jiangsu, China), anti-IL-1β (1:1,000, catalog no. ab216995, Abcam, Cambridge, United Kingdom), anti-IL-18 (1:8,000, catalog no. 10663-1-AP, Proteintech, Wuhan, China), anti-β-actin (1:5,000, catalog no. R380624, ZenBio, Chengdu, China), and anti-GAPDH (1:5,000, catalog no. R380626, ZenBio, Chengdu, China). The following day, the membranes were incubated with the appropriate HRP-conjugated secondary antibodies for 2 h. After washing, the bands were visualized with an ECL chemiluminescence substrate kit (Biosharp, Hefei, China), and quantified by densitometry using ImageJ software.

### 2.7 Immunofluorescent staining

Brain sections or cell slides fixed with 4% paraformaldehyde underwent antigen retrieval. Briefly, the prepared samples were permeabilized with 0.5% Triton X-100 for 10 min at room temperature, and blocked with 5% bovine serum albumin (BSA) for 1 h. Subsequently, they were incubated overnight at 4 °C with the following primary antibodies: anti-Iba-1 (1:300, catalog no. YM4765, Immunoway, Jiangsu, China), anti-CD86 (1:100, catalog no. YT7823, Immunoway, Jiangsu, China), and anti-CD206 (1:100, catalog no. YT5640, Immunoway, Jiangsu, China). After washing with PBST, the samples were incubated with fluorophore-conjugated secondary antibodies for 2 h, followed by nuclear staining with DAPI in the dark for 5 min. Immunofluorescence images were captured using a fluorescence microscope (Olympus IX-81/FV1000, Japan) and analyzed with ImageJ software. The whole-slide imaging was performed using CaseViewer 2.4.

### 2.8 Quantitative RT-PCR (RT-qPCR)

Total RNA extracted with TRIzol from brain tissues or treated cells. Then, complementary DNA (cDNA) was synthesized from the extracted RNA using the Revert Aid First Strand cDNA Synthesis Kit (Thermo Fisher Scientific, San Jose, CA, United States). Quantitative PCR (qPCR) was performed on a LightCycler 96 system with SYBR Green master mix (Qiagen, Germany) GAPDH was used as the endogenous control, and relative expression of target genes (TNF-α and IL-10) was determined via the 2^−ΔΔCT^ method. The primer sequences are provided in Table 1.

**TABLE 1:** Sequences of PCR primers.

Target genes	Forward primer sequence (5’→3′)	Reverse primer sequence (5’→3′)	Length (bp)
Rats-TNF-α	TGT​GGC​TCT​GGG​TCC​AAC​TC	GCA​ATC​CAG​GCC​ACT​ACT​TCA	69
Rats- IL-10	TGA​ACC​ACC​CGG​CAT​CTA​CT	CCA​AGG​AGT​TGC​TCC​CGT​TA	70
Rats-GAPDH	CAA​CTC​CCT​CAA​GAT​TGT​CAG​CAA	GGC​ATG​GAC​TGT​GGT​CAT​GA	98
Mus- TNF-α	GGT​GCC​TAT​GTC​TCA​GCC​TCT​T	GCC​ATA​GAA​CTG​ATG​AGA​GGG​AG	116
Mus- IL-10	CGG​GAA​GAC​AAT​AAC​TGC​ACC​C	CGG​TTA​GCA​GTA​TGT​TGT​CCA​GC	107
Mus-GAPDH	CAT​CAC​TGC​CAC​CCA​GAA​GAC​TG	ATG​CCA​GTG​AGC​TTC​CCG​TTC​AG	130

### 2.9 Cell culture

BV2 cells were obtained from the Cell Bank of the Chinese Academy of Sciences (Shanghai, China). The cells were routinely cultured in high-glucose Dulbecco’s Modified Eagle Medium (DMEM) supplemented with 10% fetal bovine serum (FBS) and 1% penicillin-streptomycin, and maintained at 37 °C in a humidified atmosphere of 5% CO_2_. All experiments were performed using cells in the logarithmic growth phase.

### 2.10 Cell viability

BV2 cells (1 × 10^4^/well) were seeded in 96-well plates and allowed to adhere for 24 h. To assess the effect of S-GLSP on cell viability, the cells were treated with a range of S-GLSP concentrations (0–175 μg/mL) for 24 h. In a separate experiment designed to evaluate the protective effect of S-GLSP against LPS-induced toxicity, cells were pretreated with S-GLSP (25, 50, 75 μg/mL) for 4 h, after which all groups except the control (CTRL) were exposed to 1 μg/mL LPS for an additional 24 h. Following the respective treatments, 20 μL of MTT solution (5 mg/mL, Solarbio, M1020) was added to each well, and the plates were incubated at 37 °C for 4 h. The medium was then carefully aspirated, and the resulting formazan crystals were dissolved in 150 μL of dimethyl sulfoxide (DMSO). Absorbance was measured at 490 nm using a microplate reader (Peiou, Shanghai, China).

### 2.11 Transfection

BV2 cells were transfected with 50 nM NLRP3 siRNA (Hanheng Biotechnology Co., Wuhan, China) using Lipofectamine™ 2000 (Thermo Fisher Scientific, 11668019) for 6 h prior to drug treatment. A non-targeting siRNA (NC) was used as negative control. Following transfection, the cells were divided into six experimental groups: CTRL, LPS, LPS + S-GLSP, LPS + NC, LPS + si-NLRP3, and LPS + si-NLRP3 + S-GLSP for subsequent investigations.

### 2.12 EdU staining

Cell proliferation was assessed using the EdU Cell Proliferation Detection Kit (Sangon Biotech, Shanghai, China) according to the manufacturer’s protocol. After the staining procedure, fluorescence images were acquired, and the EdU-positive cell ratio was quantified using ImageJ software for subsequent statistical analysis.

### 2.13 Statistical analysis

All data were analyzed with SPSS 26.0 and are expressed as the mean ± standard error of the mean (SEM). Differences among groups were evaluated by one-way analysis of variance (ANOVA), followed by Dunnett’s *post hoc* test. In cases where the assumption of homogeneity of variance was met, the least significant difference (LSD) test was applied. A P-value of less than 0.05 was considered statistically significant.

## 3 Results

### 3.1 The main chemical compounds of S-GLSP extract

**FIGURE 2:**
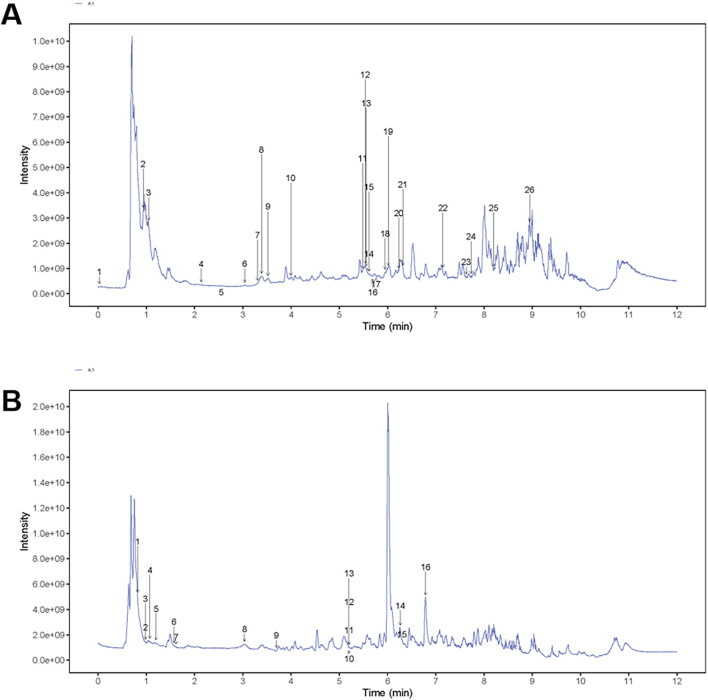
Total ion diagram of S-GLSP extract in positive and negative ion mode. **(A)** NEG; **(B)** POS.

UHPLC analysis was employed to characterize the chemical profile of the S-GLSP extract. As shown in the total ion chromatogram (TIC) in positive ion mode (Figure 2), a total of 42 compounds were identified, which were classified into flavonoids, alkaloids, terpenoids, saccharides, phenolics, fatty acids, nucleosides, amino acids, and other compound classes (Table 2).

**TABLE 2:** Chemical compounds analysis of S-GLSP extract by UHPLC.

NO.	Type	Compound	Molecular formula	tR (s)	Molecular ion (m/%)
1	NEG	Glucose	C6H12O6	1.4	161.0448
2	NEG	7-Methylxanthine	C6H6N4O2	55.8	165.0396
3	NEG	Malonic acid	C3H4O4	62.7	103.0032
4	NEG	Methylsuccinic acid	C5H8O4	128.2	131.0344
5	NEG	Thymidine	C10H14N2O5	153.2	241.082
6	NEG	Vidarabine	C10H13N5O4	182.2	266.0883
7	NEG	D-Sorbitol	C6H14O6	198.1	181.071
8	NEG	3-Furoic acid	C5H4O3	203.1	111.0083
9	NEG	Succinyladenosine	C14H17N5O8	211.2	382.0987
10	NEG	Ketoleucine	C6H10O3	239.5	129.0551
11	NEG	(6aR,11aR)-3-methoxy-6a,11a-dihydro-6H-benzofuro [3,2-c]chromene-4,9-diol	C16H14O5	328.8	285.0756
12	NEG	[(2R,3R,4R,8S,10R,11R)-2,3,11-trihydroxy-4,6,6,10-tetramethyl-8-tricyclo [5.3.1.04,11]undecanyl] acetate	C30H42O8	332.2	529.2781
13	NEG	[(2R,3R,4R,8S,10R,11R)-2,3,11-trihydroxy-4,6,6,10-tetramethyl-8-tricyclo [5.3.1.04,11]undecanyl] acetate	C17H28O5	333.5	311.185
14	NEG	Sodium 3-(3,4-dihydroxyphenyl)-2-hydroxypropanoate	C9H10O5	336.6	197.0446
15	NEG	3-(3,4-Dihydroxyphenyl)-2-hydroxypropanoic acid	C9H10O5	336.6	197.0446
16	NEG	Ganoderic acid I	C30H44O8	341.4	531.2938
17	NEG	(2R,6R)-2-methyl-4-oxo-6-[(3S,5R,7S,10S,13R,14R,15S,17R)-3,7,15-trihydroxy-4,4,10,13,14-pentamethyl-11-oxo-1,2,3,5,6,7,12,15,16,17-decahydrocyclopenta [a]phenanthren-17-yl]heptanoic acid	C30H46O7	345.4	517.3141
18	NEG	4-Chromanone	C9H8O2	356.9	147.0445
19	NEG	Ganoderic acid B	C30H44O7	360.7	515.2988
20	NEG	6-(7-hydroxy-4,4,10,13,14-pentamethyl-3,11,15-trioxo-1,2,5,6,7,12,16,17-octahydrocyclopenta [a]phenanthren-17-yl)-2-methyl-4-oxo-hept-5-enoic acid	C30H40O7	373.8	493.2567
21	NEG	(4R)-4-[(5R,7S,10S,13R,14R,17R)-7-hydroxy-4,4,10,13,14-pentamethyl-3,11,15-trioxo-1,2,5,6,7,12,16,17-octahydrocyclopenta [a]phenanthren-17-yl]pentanoic acid	C27H38O6	378.9	457.2574
22	NEG	5-[2-(3-furyl)ethyl]-8-hydroxy-5,6,8a-trimethyl-3,4,4a,6,7,8-hexahydronaphthalene-1-carboxylic acid	C20H28O4	428.8	331.1899
23	NEG	(E)-5-(2,3-dimethyl-3-tricyclo [2.2.1.02,6]heptanyl)-2-methyl-pent-2-enoic acid	C15H22O2	457.7	233.1536
24	NEG	2,4-bis(3-methylbut-2-enyl)-6a,11a-dihydro-6H-benzofuro [3,2-c]chromene-3,9-diol	C25H28O4	463.7	391.1872
25	NEG	3,5-Di-tert-butylphenol	C14H22O	491.8	205.1587
26	NEG	Myristic acid	C14H28O2	536.5	227.2003
1	POS	Isonicotinic acid	C6H5NO2	49	124.0388
2	POS	6-(hydroxymethyl)pyridin-3-ol	C6H7NO2	58.7	126.0544
3	POS	Methyl (2S)-5-oxopyrrolidine-2-carboxylate	C6H9NO3	58.7	126.0544
4	POS	Uridine 5′-monophosphate (UMP)	C9H13N2O9P	64.3	325.0421
5	POS	Adenine	C5H5N5	72.1	136.0613
6	POS	Guanosine	C10H13N5O5	94.3	284.0979
7	POS	Normetanephrine	C9H13NO3	96.9	166.0856
8	POS	Adenosine	C10H13N5O4	182	268.1027
9	POS	3-Hydroxypyridine	C5H5NO	221.8	96.044
10	POS	3,5-dihydroxy-2-(4-hydroxyphenyl)-7-[(2S,3R,4S,5S,6R)-3,4,5-trihydroxy-6-(hydroxymethyl)tetrahydropyran-2-yl]oxy-chromen-4-one	C21H20O11	312.1	449.1064
11	POS	Rhodionin	C21H20O11	312.1	449.1064
12	POS	2-(2,6-dihydroxyphenyl)-3,5,7-trihydroxy-chromone	C15H10O7	312.1	303.0488
13	POS	Quercetin	C15H10O7	312.1	303.0488
14	POS	Ganoderic acid A	C30H44O7	375.9	499.3032
15	POS	Ganoderic acid F	C32H42O9	377.9	571.2877
16	POS	Sphingosine	C18H37NO2	407.4	282.2779

### 3.2 Neuroprotective effect of S-GLSP in Alzheimer’s disease rats

**FIGURE 3:**
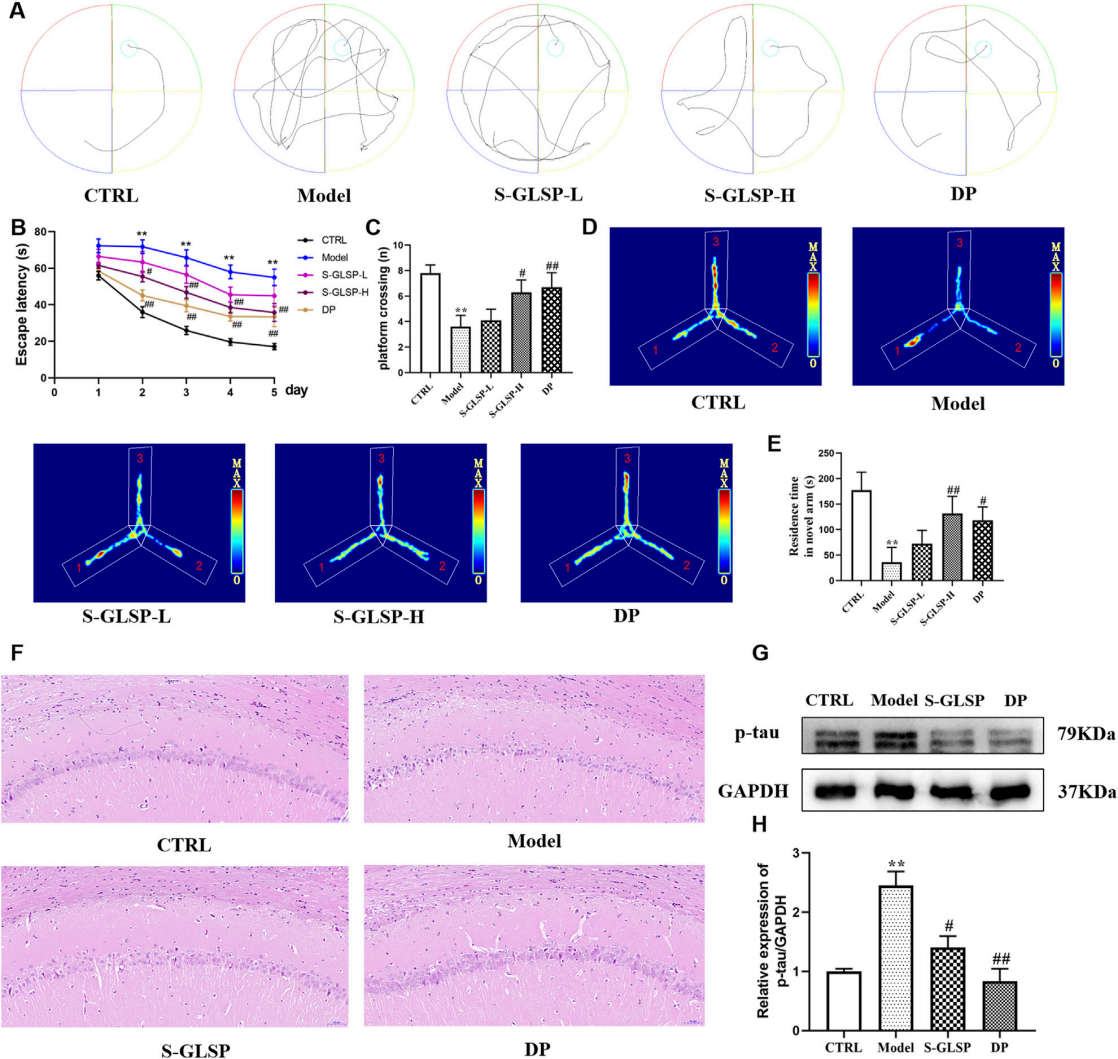
S-GLSP ameliorates neuronal damage and Alzheimer’s disease-specific pathology in AD model rats. (n = 10). **(A)** The swimming trajectory map on the fifth day of the MWM test. **(B)** The escape latency to find the hidden platform of the Morris water maze test (days 1–5). **(C)** The number of platform crossings of the Morris water maze test. **(D)** The motion trajectory diagram of the Y-maze test. **(E)** The time spent in the novel arm in Y-maze test. **(F)** H&E staining of hippocampal CA1 region (scale bar = 50 μm). **(G–H)** The protein levels of p-tau in the brain lysates were assessed by WB. Data are shown as the mean ± SEM. *P < 0.01 vs. CTRL; ^#^ *P* < 0.05, ^##^ *P* < 0.01 vs. Model.

Behavioral assessments demonstrated that S-GLSP treatment at 720 mg/kg significantly improved cognitive performance in AD model rats. In the Morris water maze test, S-GLSP markedly shortened escape latency and increased the number of platform crossings (Figures 3A–C). Furthermore, the five-day learning curves revealed a substantial improvement in learning ability in the S-GLSP-H and DP groups compared to the Model group. Consistently, in the Y-maze test, S-GLSP significantly prolonged the time spent in the novel arm (Figures 3D,E). Based on these results, the 720 mg/kg dose was selected for subsequent mechanistic studies.

To evaluate both general neuronal morphology and specific Alzheimer’s disease pathology, we performed H&E staining and assessed the levels of hyperphosphorylated tau (p-tau), a core pathological protein in AD. Histopathological evaluation by H&E staining revealed severe neuronal damage in the hippocampus of model group rats, including disordered arrangement, cell loss, and structural disintegration. In contrast, S-GLSP treatment notably ameliorated these pathological changes, yielding a neuronal morphology comparable to the donepezil-treated group (Figure 3F). Given that hyperphosphorylation of tau protein is a hallmark neuropathological feature of AD (Azarpazhooh et al., 2020), we further examined the effect of S-GLSP on tau phosphorylation. Western blot analysis confirmed that S-GLSP significantly suppressed the expression of phosphorylated tau (p-tau) (Figures 3G,H), indicating its ability to modulate a key AD-related pathogenic process. We all know that the overexpression of phosphorylated tau (p-tau) protein, which forms neurofibrillary tangles, is one of the pathological hallmarks of AD (Azarpazhooh et al., 2020). Therefore, the ability to inhibit tau protein hyperphosphorylation is a key criterion for evaluating potential AD drug treatments. In our experiments, treatment with S-GLSP notably ameliorated both the structural neuronal damage observed by H&E staining and the aberrant p-tau accumulation, indicating its dual protective role against both general neurotoxicity and specific AD-related pathogenic processes.

### 3.3 Effect of S-GLSP on microglial polarization in Alzheimer’s disease rats

**FIGURE 4:**
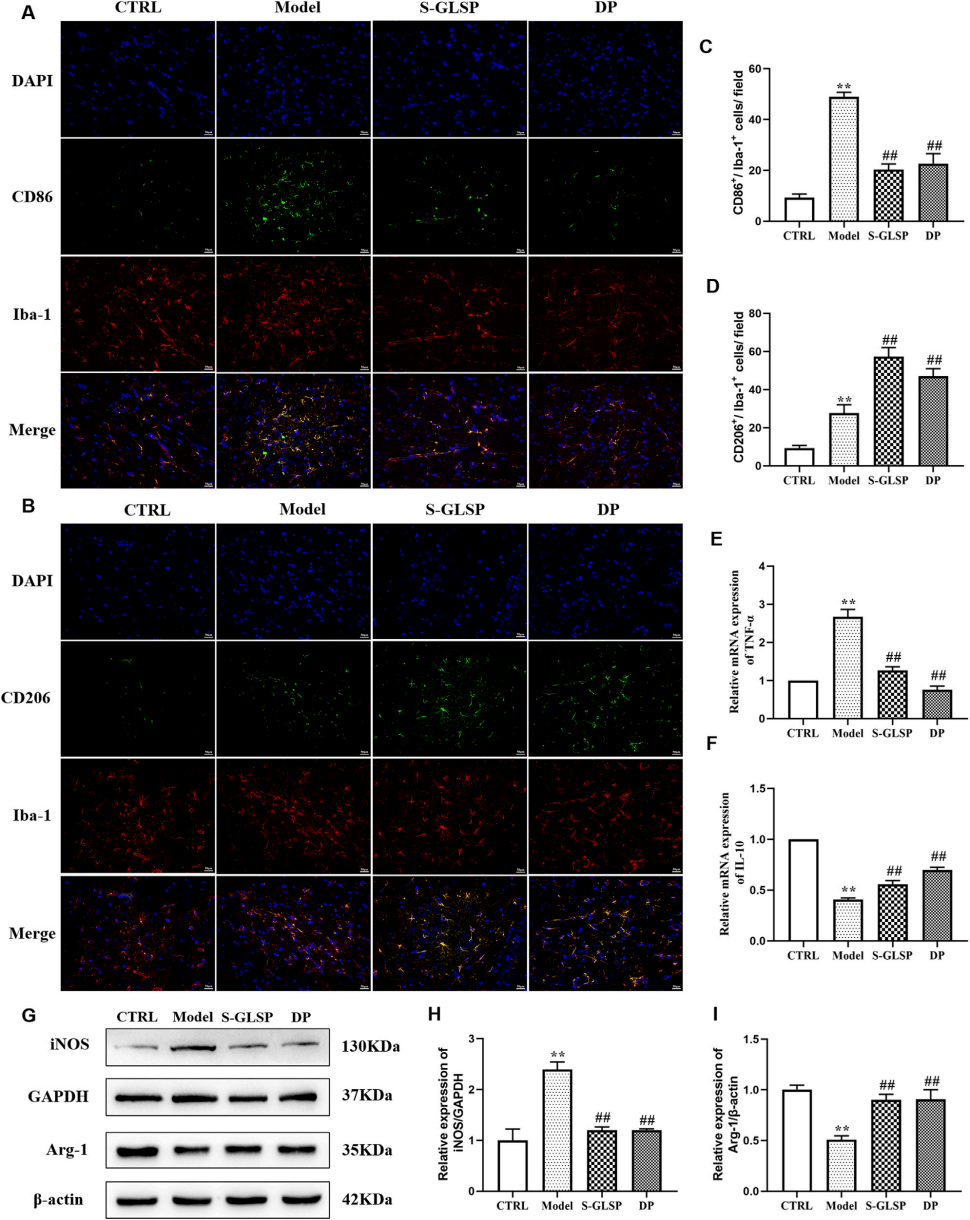
S-GLSP promotes microglial polarization from M1 to M2 phenotype in AD rats. (n = 3). **(A,B)** Representative immunofluorescence images showing colocalization of Iba-1 (red) with CD86 (green, M1 marker) or CD206 (green, M2 marker). (scale bar = 50 μm). **(C,D)** Quantification analysis of CD86^+^/Iba-1^+^ and CD206^+^/Iba-1^+^ colabeled cells across different groups. **(E,F)** The mRNA levels of TNF-α (M1 marker) and IL-10 (M2 marker). **(G–I)** The protein levels of iNOS and Arg-1. Data are shown as the mean ± SEM. *P < 0.01 vs. CTRL; ^#^ *P* < 0.05, ^##^ *P* < 0.01 vs. Model.

Microglial overactivation contributes to sustained neuroinflammation, which in turn accelerates Alzheimer’s disease progression (Villegas-Llerena et al., 2016). Specifically, excessive polarization toward the M1 phenotype promotes neuronal injury and degeneration (Subhramanyam et al., 2019), whereas M2 microglia exert neuroprotective effects by clearing pathogenic debris and releasing anti-inflammatory and neurotrophic mediators (Ginhoux et al., 2010; Colonna and Butovsky, 2017). In this study, immunofluorescence analysis revealed that S-GLSP (720 mg/kg) significantly decreased the density of M1 microglia (CD86^+^/Iba-1^+^) while increasing that of M2 microglia (CD206^+^/Iba-1^+^) (Figures 4A–D). Consistent with these findings, Western blot results showed that S-GLSP downregulated the M1 marker iNOS and upregulated the M2 marker Arg-1 (Figures 4G–I). Further support came from RT-qPCR analysis, which indicated that S-GLSP enhanced mRNA expression of the anti-inflammatory cytokine IL-10 and suppressed that of the pro-inflammatory cytokine TNF-α (Figures 4E,F).

### 3.4 Effect of S-GLSP on NLRP3 inflammasome activation in AD rats

**FIGURE 5:**
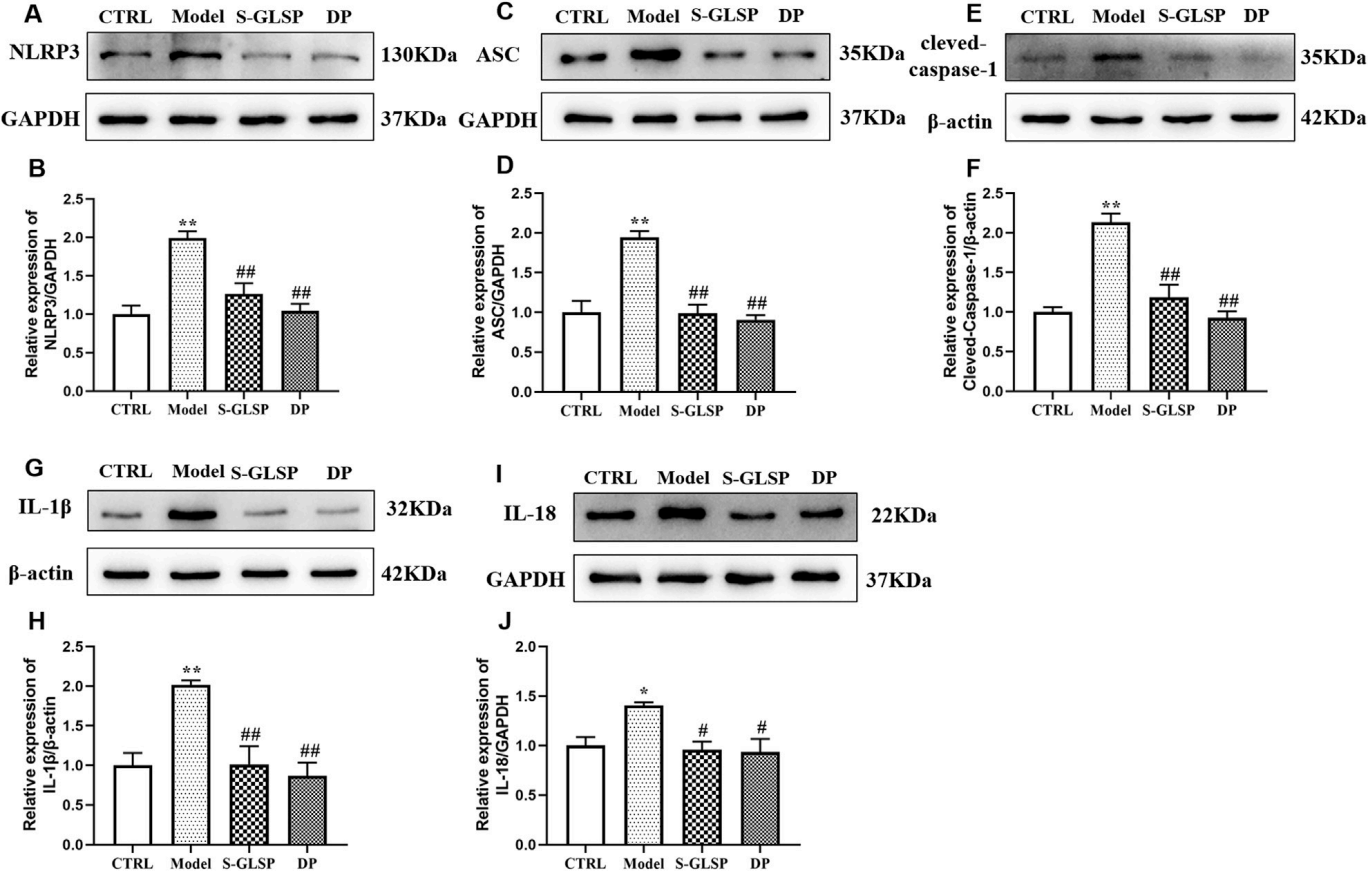
S-GLSP suppresses NLRP3 inflammasome activation in AD rats. (n = 3). **(A–J)** The Protein levels of NLRP3, ASC, cleved-caspase-1, IL-1β and IL-18. Data are shown as the mean ± SEM. *P < 0.01 vs. CTRL; ^#^ *P* < 0.05, ^##^ *P* < 0.01 vs. Model.

The NLRP3 inflammasome plays a pivotal role in Alzheimer’s disease by regulating neuroinflammatory responses (Feng et al., 2020; Jha et al., 2024). Its activation in microglia can be triggered by aggregated Aβ oligomers and fibrils, which act as damage-associated molecular patterns (DAMPs) (Ayyubova and Madhu, 2025). Consistent with these findings, our Western blot analysis showed that the protein levels of NLRP3, ASC, and cleaved-caspase-1 were significantly increased in the brains of AD model rats (Figures 5A–F). This inflammasome activation was accompanied by elevated expression of the downstream inflammatory cytokines IL-1β and IL-18 (Figures 5G–J). Notably, treatment with S-GLSP significantly suppressed the upregulation of these inflammasome-related proteins and cytokines (Figures 5A–J).

### 3.5 Effect of S-GLSP on LPS-induced BV-2 microglial cells

**FIGURE 6:**
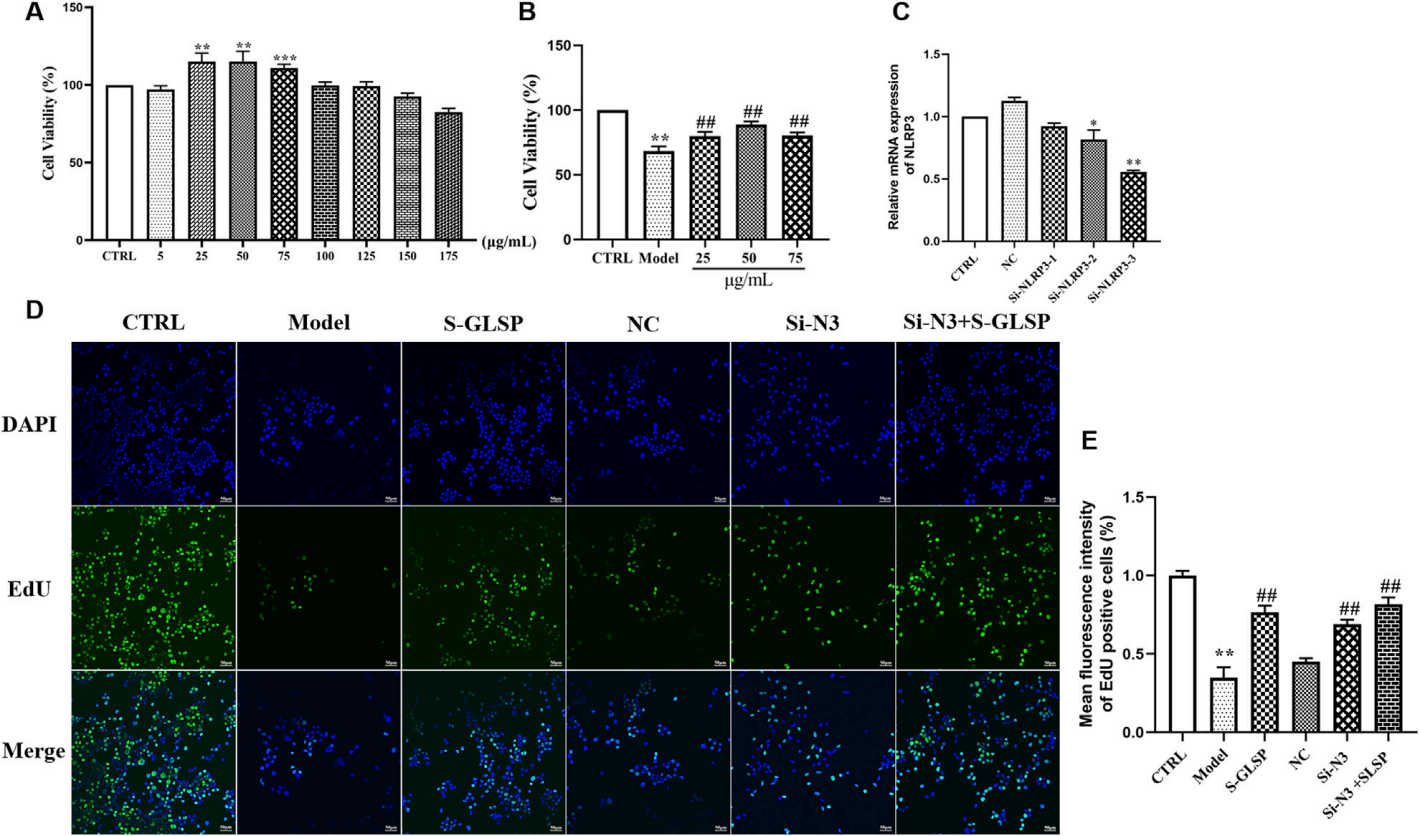
S-GLSP protects against LPS-induced injury in BV2 microglial cells. (n = 3). **(A)** Cell viability after 24 h treatment with different concentrations of S-GLSP. **(B)** Cell viability following S-GLSP pretreatment and subsequent LPS challenge. **(C)** NLRP3 mRNA expression after siRNA transfection. **(D,E)** Cell proliferation assessed by EdU staining. Data are shown as the mean ± SEM. *P < 0.01 vs. CTRL; ^#^ *P* < 0.05, ^##^ *P* < 0.01 vs. Model.

Based on this, the safe concentration range was determined to be 25–75 μg/mL. In LPS-stimulated BV-2 cells, viability was significantly reduced; however, pretreatment with S-GLSP (25, 50, and 75 μg/mL) attenuated this decrease in a concentration-dependent manner, with the most pronounced effect observed at 50 μg/mL (Figure 6B). This concentration was therefore selected for subsequent experiments.

To explore the role of NLRP3 in the anti-AD mechanism of S-GLSP, we performed NLRP3 knockdown in BV-2 cells. Among three siRNA constructs, si-NLRP3-3 achieved the highest knockdown efficiency and was used in further studies (Figure 6C). EdU staining revealed that LPS suppressed BV-2 cell proliferation, an effect that was reversed by either NLRP3 silencing or S-GLSP treatment. Furthermore, the combination of NLRP3 knockdown and S-GLSP treatment showed an additive, but not statistically synergistic, trend towards further enhancing cell proliferation compared to either intervention alone (Figures 6D,E).

### 3.6 Effect of S-GLSP on LPS-induced microglial polarization in BV-2 cells

**FIGURE 7:**
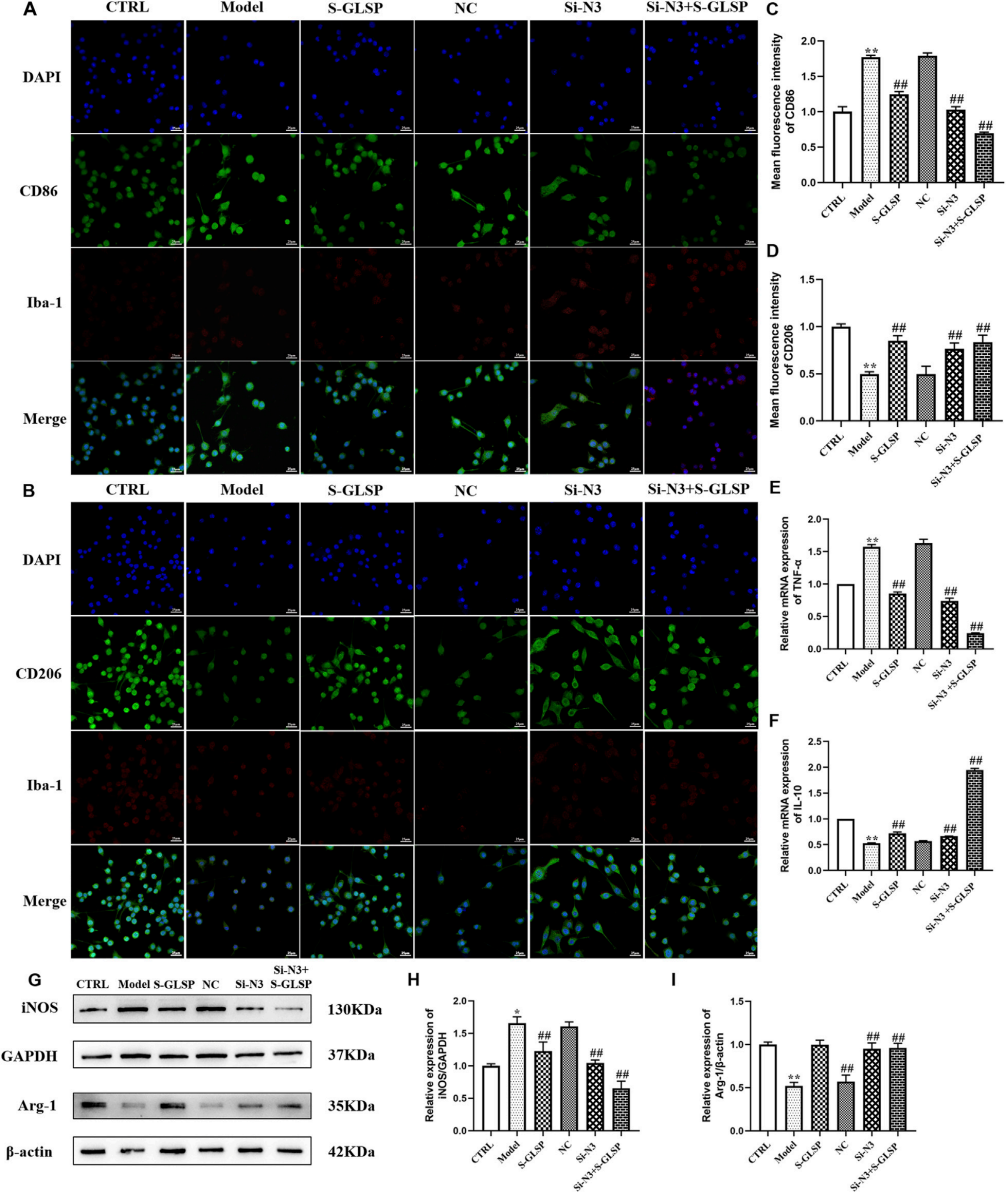
S-GLSP promotes M2 microglial polarization in LPS-induced BV2 cells. (n = 6). **(A,B)** Representative immunofluorescence images showing Iba-1 (red) colocalized with CD86 (green) or CD206 (green). (Scale bar = 25 μm). **(C,D)** Quantification of CD86^+^ and CD206^+^ colabeled cells across different groups. **(E,F)** The mRNA levels of TNF-α and IL-10 in LPS-induced BV2 cells. **(G–I)** The protein levels of iNOS and Arg-1in LPS-induced BV2 cells. Data are shown as the mean ± SEM. *P < 0.01 vs. CTRL; ^#^ *P* < 0.05, ^##^ *P* < 0.01 vs. Model.

Immunofluorescence and Western blot analyses demonstrated that LPS stimulation significantly promoted microglial polarization toward the M1 phenotype, as evidenced by increased expression of M1 markers and decreased expression of M2 markers (Figures 7A–D,G-I). Both NLRP3 knockdown and S-GLSP treatment effectively reversed this LPS-induced polarization shift. Moreover, the combination of NLRP3 silencing and S-GLSP treatment produced a synergistic effect, more potently restoring the M1/M2 balance (Figures 7A–D,G-I).

Consistent with these findings, RT-qPCR analysis revealed that LPS stimulation significantly upregulated TNF-α mRNA expression and downregulated IL-10 expression. NLRP3 knockdown and S-GLSP treatment each attenuated these changes, while their combination resulted in a more pronounced anti-inflammatory response (Figures 7E,F).

### 3.7 Effect of S-GLSP on NLRP3 inflammasome activation in LPS-induced BV-2 cells

**FIGURE 8:**
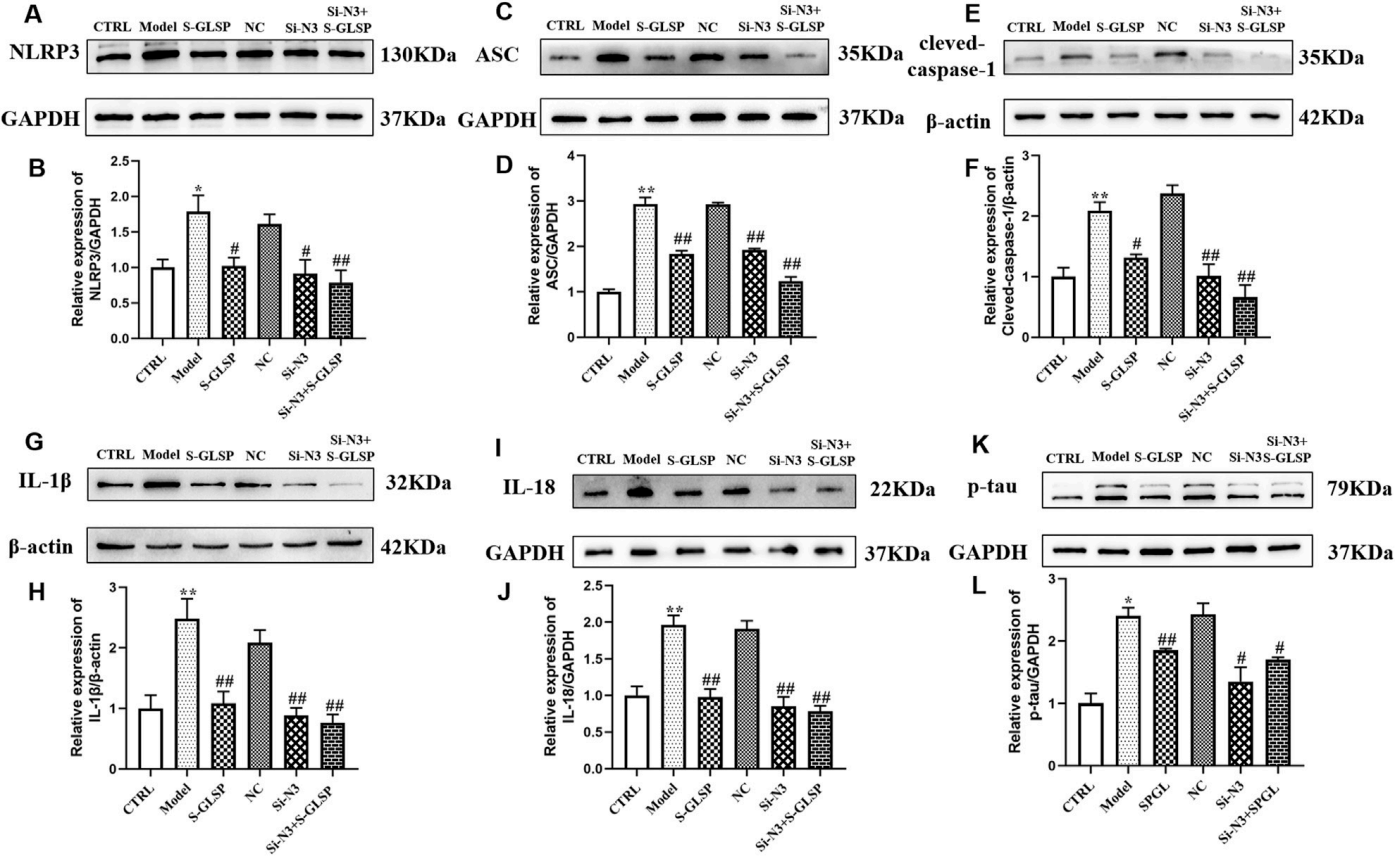
S-GLSP inhibits NLRP3 inflammasome activation in LPS-induced BV2 cells. (n = 3). **(A–L)** The Protein levels of NLRP3, ASC, cleved-caspase-1, IL-1β, IL-18 and p-tau. Data are shown as the mean ± SEM. *P < 0.01 vs. CTRL; ^#^ *P* < 0.05, ^##^ *P* < 0.01 vs. Model.

Western blot analysis demonstrated that LPS stimulation significantly upregulated the expression of NLRP3 inflammasome compounds, including NLRP3, ASC, and cleaved-caspase-1, in BV-2 cells (Figures 8A–F). Consistent with inflammasome activation, the levels of the downstream inflammatory cytokines IL-1β and IL-18 were also elevated (Figures 8G–J). Both NLRP3 knockdown and S-GLSP treatment effectively suppressed these changes, and their combination resulted in a synergistic inhibition of the NLRP3 inflammasome pathway.

To further evaluate the functional relevance of these findings, we examined neuronal tau pathology in HT-22 cells following co-culture with the treated BV-2 cells. Western blot analysis revealed that S-GLSP significantly reduced the expression of phosphorylated tau (p-tau) in HT-22 cells exposed to conditioned media from LPS-stimulated BV-2 cells (Figures 8K,L). This protective effect suggests that S-GLSP mitigates tau hyperphosphorylation through mechanisms involving the suppression of NLRP3 inflammasome activation, regulation of microglial polarization, and inhibition of pro-inflammatory cytokine release.

## 4 Discussion

The principal finding of this study is that S-GLSP confers remarkable protection against neuroinflammation in Alzheimer’s disease models by modulating microglial polarization from a detrimental M1 state towards a protective M2 state. Crucially, we have identified that the underlying mechanism involves the suppression of the NLRP3 inflammasome pathway, a key driver of neuroinflammatory processes. These results not only underscore the therapeutic potential of S-GLSP for AD but also provide a mechanistic elucidation for its traditional use in promoting neurological health.

Alzheimer’s disease (AD) pathogenesis involves a complex interplay of pathological mechanisms, with neuroinflammation increasingly recognized as a critical driver of disease progression. Elevated levels of neuroinflammatory mediators have been consistently detected in the cerebrospinal fluid, blood, and cerebral cortex of AD patients (Swardfager et al., 2010; Leng and Edison, 2021). Central to this neuroinflammatory process are microglia, the resident immune cells of the central nervous system that maintain brain homeostasis through inflammation regulation, phagocytic clearance, and neural support (Singh, 2022; Gao et al., 2023). In response to pathological stimuli such as amyloid-beta (Aβ) and neurofibrillary tangles (NFTs) accumulation, microglia undergo activation and polarize into distinct functional phenotypes (Kwon and Koh, 2020; Kulkarni et al., 2022; Botella Lucena and Heneka, 2024). The classical M1 phenotype exhibits neurotoxic properties through the release of pro-inflammatory cytokines including TNF-α, IL-1β, and IL-6, which accelerate neurodegenerative processes and contribute to neuronal damage (Ogunmokun et al., 2021; Kulkarni et al., 2022). Conversely, the alternative M2 phenotype demonstrates neuroprotective effects by secreting anti-inflammatory factors such as IL-4, IL-10, and TGF-β, which help regulate immune responses and promote tissue repair (Jiang et al., 2021). Repolarization of microglia from the M1 to the M2 phenotype has emerged as a promising therapeutic strategy for Alzheimer’s disease. In AD, the anti-inflammatory M2 phenotype is often compromised, leading to a polarization imbalance that exacerbates neuroinflammation and neuronal injury (Zhou et al., 2020; Amanollahi et al., 2023). Supporting this concept, studies have demonstrated that promoting M2 polarization-for instance, through TLR4 inhibition in APP/PS1 mice-exerts neuroprotective effects and ameliorates cognitive deficits (Cui et al., 2020). More broadly, enhancing M2 microglial activity, whether by pharmacological or genetic approaches, has been shown to attenuate neuroinflammation, improve phagocytic clearance of plaques and tangles, and restore brain homeostasis (Merighi et al., 2022). In line with existing literature, our study confirmed that AD model rats exhibit a marked microglial polarization imbalance, characterized by an upregulation of M1 markers (CD86, iNOS) and a concomitant downregulation of M2 markers (CD206, Arg-1). This skewed polarization towards the M1 phenotype fosters a chronic inflammatory milieu, contributing to neuronal damage and the cognitive deficits observed in our models. Consistent with our other findings, further serological tests revealed a significant increase in the pro-inflammatory cytokine TNF-α and a concurrent decrease in the anti-inflammatory cytokine IL-10 in the serum of model group rats. The efficacy of S-GLSP in rectifying this imbalance-effectively promoting an M2-dominant anti-inflammatory profile. This finding is consistent with the growing therapeutic strategy of targeting microglial polarization to halt disease progression.

Ganoderma lucidum (Lingzhi) has been valued in traditional Chinese medicine for millennia due to its therapeutic properties. Ganoderma spores, regarded as the essence of the mushroom, contain its complete genetic material and are now widely used as a medicinal and nutraceutical product thanks to advances in spore wall-breaking technology (Shen et al., 2020). Chemical analyses have confirmed that S-GLSP contains a wide spectrum of bioactive compounds, including flavonoids, alkaloids, terpenoids, saccharides, phenolics, fatty acids, nucleosides, amino acids, and phenylpropanoids. These compounds contribute to its neuroprotective effects in AD through multiple pathways. For instance, Ganoderma triterpenoids have been shown to inhibit the ROCK signaling pathway, reducing neuronal damage and apoptosis in the hippocampus and improving cognitive deficits in APP/PS1 mice (Yu et al., 2020). Similarly, Ganoderic acid A (GAA) alleviates neuroinflammation and enhances cognitive function by modulating the Th17/Treg axis (Zhang et al., 2021), while Ganoderma lucidum polysaccharides suppress the secretion of pro-inflammatory mediators such as inducible nitric oxide synthase (iNOS), interleukin-6 (IL-6), and interleukin-1 beta (IL-1β), thereby attenuating microglia-mediated neuroinflammation (Cai et al., 2017; Liu et al., 2023; Chen et al., 2025). Collectively, these studies underscore the indisputable anti-inflammatory and neuroprotective properties of Ganoderma lucidum and its bioactive constituents (Cao et al., 2024). In line with these findings, the present study demonstrates that S-GLSP treatment significantly ameliorates learning and memory impairments in AD rats, supporting its potential as a therapeutic candidate for AD. This observation is consistent with a previous report that S-GLSP improves cognitive function in a rat model of sporadic AD by inhibiting the NF-κB/NLRP3 inflammatory pathway in the medial prefrontal cortex (Qin et al., 2024). Moreover, our *in vitro* and *in vivo* experiments revealed that S-GLSP not only mitigates neuronal injury but also promotes the transition of microglia from the pro-inflammatory M1 phenotype to the anti-inflammatory M2 phenotype, providing mechanistic insight into its protective role in AD.

Furthermore, our results indicate that S-GLSP significantly suppresses both NLRP3 inflammasome expression and the secretion of its downstream inflammatory cytokines, IL-1β and IL-18. The NLRP3 inflammasome, a multiprotein complex comprising NLRP3, ASC, and pro-caspase-1 (Abderrazak et al., 2015), is abundantly expressed in the central nervous system, particularly in microglia. Upon activation, it triggers the cleavage of pro-caspase-1 into its active form, caspase-1, which subsequently processes pro-IL-1β and pro-IL-18 into their mature forms, driving neuroinflammatory responses (Tschopp and Schroder, 2010; Heneka et al., 2018; Van Zeller et al., 2021). Evidence from neuropathological examinations and transgenic models underscores the pivotal role of NLRP3 in AD progression, facilitating the accumulation of β-amyloid and tau pathology (Pereira et al., 2019). Importantly, the release of IL-1β and IL-18 reinforces microglial activation toward the pro-inflammatory M1 phenotype, creating a feed-forward cycle of inflammation. In this study, we demonstrated that S-GLSP effectively disrupts this cascade by inhibiting NLRP3 inflammasome activation and modulating microglial polarization, as confirmed in both *in vitro* and *in vivo* experiments. Notably, we utilized a high-dose LPS stimulation paradigm in BV2 microglia, which resulted in the full activation of the NLRP3 inflammasome, as evidenced by the cleavage of caspase-1 and the maturation of IL-1β and IL-18. While this deviates from the canonical two-signal model often observed in other cell types, it is consistent with reports of signal integration in microglia under potent inflammatory stress (Yang et al., 2023; Gu et al., 2024). This context underscores the significance of our finding that S-GLSP effectively disrupts this complete activation cascade.

**FIGURE 9:**
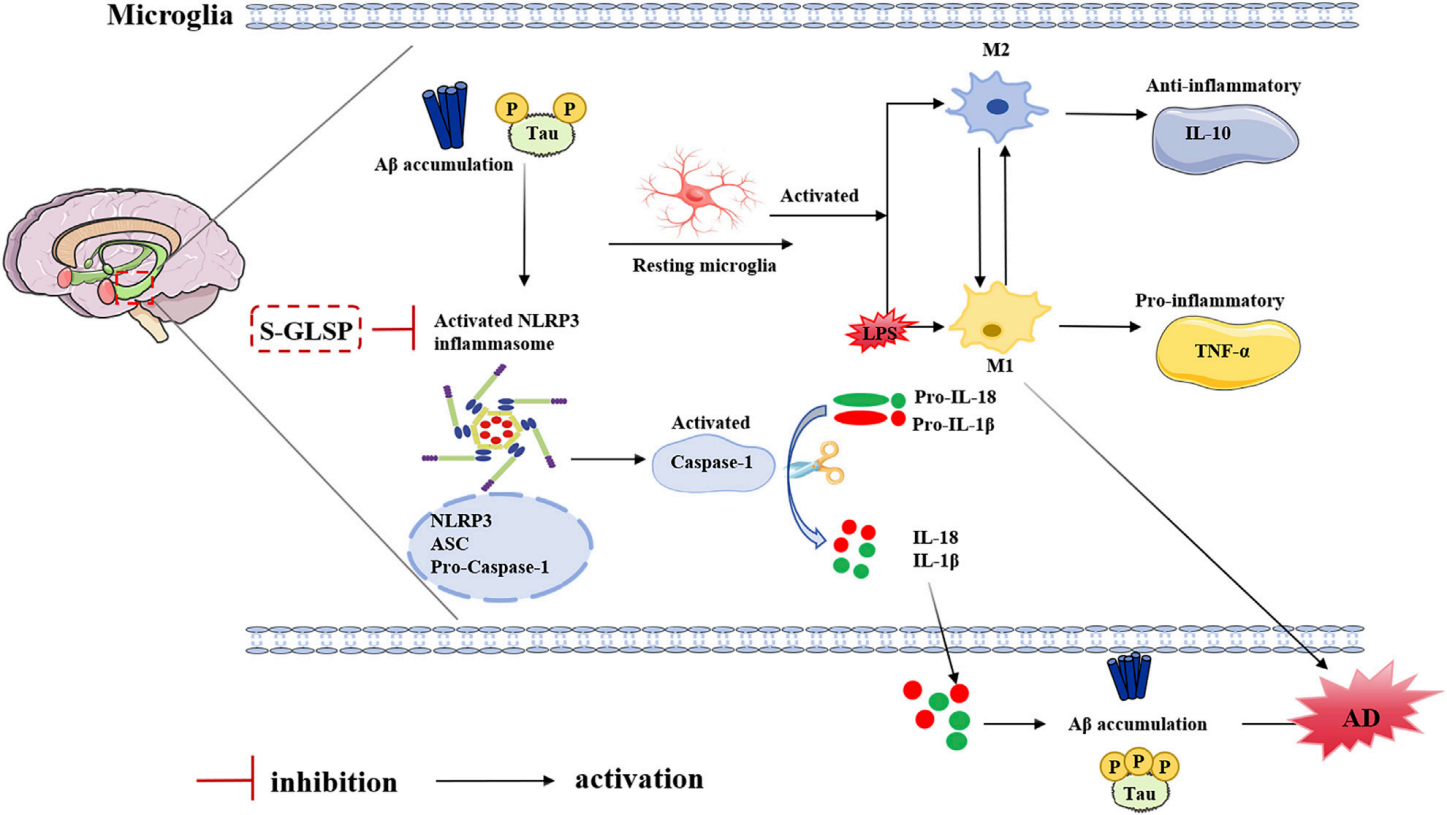
Proposed mechanism: S-GLSP promotes M2 microglial polarization by regulating NLRP3 inflammasome activation.

In conclusion, our study provides compelling evidence that S-GLSP is a promising natural product for mitigating AD-related neuroinflammation. We have mechanistically linked its therapeutic effects to the inhibition of the NLRP3 inflammasome, which in turn rebalances microglial polarization, thereby creating a neuroprotective environment. A detailed diagram of this mechanism is shown in Figure 9. These findings scientifically validate the potential of S-GLSP as a viable candidate for developing innovative AD therapeutics. Future research should focus on isolating the most active anti-inflammatory fractions within S-GLSP and validating these effects in advanced models and, ultimately, in clinical trials.

## Data Availability

The original contributions presented in the study are included in the article/Supplementary Material, further inquiries can be directed to the corresponding authors.
